# Supplementary material for: A clinical protocol for a German birth cohort study of the Maturation of Immunity Against respiratory viral Infections (MIAI)
Source: Front Immunol. 2024 Sep 17;15:1443665. doi: 10.3389/fimmu.2024.1443665 (PMC11442434; doi:10.3389/fimmu.2024.1443665)
Supplement: Supplementary file 1 [file Table1.docx]

**SOP MIAI – Clinical management inpatient and outpatient follow-up**

**I. Test subjects**

Inclusion criteria:

- Term > 37 +0 - 41 +6 weeks' gestation
- Place of residence of the family in the city or district of Würzburg
- Birth in the University Hospital of Würzburg (UKW)
- Consent of both parents is available; in the case of single parents, consent of the custodial parent (see Parents‘ information form, informed consent form)

Exclusion criteria:

- Congenital immunological, metabolic disease, malformation
- Triple I (intrauterine inflammation or infection)
- Early onset neonatal sepsis
- Foreseen change of residence to outside the city or district of Würzburg in the next 12 months

**II. Preparations / General information**

1. **recruitment:**

Introduction to the study at information evenings for becoming parents

Study flyer dissemination in UKW birth mode outpatient clinic or hospital admission for delivery

Homepage ([www.ukw.de/miai](http://www.ukw.de/miai)) -> Online form

1. **Screening**:

**a)** Daily review of births -> screening log with potential study participants who meet the inclusion criteria. Number of potential study participants is noted on the whiteboard.

1. Approaching potential study participants by study nurse -> handing out patient parent information & flyer

Direct refusal to participate in the study -> Revise the number of potential study participants on the whiteboard

The study nurse makes a note in the screening log.

**III. Sampling d0 - Delivery room**

- Collection of umbilical cord blood by a midwife attending the birth
- Collection of **umbilical cord blood** and birth certificates every working day in the morning by the laboratory team
- Checking the inclusion criteria and the quantity and quality of the sample
- Processing of the sample is noted in the screening log
- Written informed consent is obtained, digitized and archived with the birth certificate by the study doctor
- Entry of the sample and metadata in the metadata list for participants, if parents refuse to participate at this stage collected material will be discarded

**IV. Physical examination and sampling d1-3 on the ward**

- Study nurse inquires about study **participation** after parents were **informed** about the study the day before
- Consent is digitized and archived with birth sheet
- Contact details of the parents are noted and follow-up appointments are entered in the calendar
- **Initial physical examination and sampling at d1-3** is carried out by the study physician after study approval:
  a) either immediately after information (if the mother wants to leave the clinic before regular check up (U2, from 48h to 10 days after birth)
  b) or as part of the U2 in a women's clinic.
- Study participation packages for sampling are prepared by Study Nurse for sampling at different follow-up examinations
- **Biosamples at d1-3** (order: swabs, nasal brush, BE, MM, stool): - 2x skin swabs (sternum) – moisten swab for swab A in submitted PBS+BSA, swab sternum and in Eppendorf tube with PBS+BSA; Swab for swab B from moistened skin on sternum and then dry in Eppendorf tube
  - 3x throat swab – Swab A in Eppendorf tube with PBS+BSA presented, swabs B and C in an Eppendorf tube
  - Nasal brush (1 brush per nostril -> put both in the same prepared 5ml Eppendorf tube)
  - Serum (min. 0,5ml)
  - EDTA-Blood (min. 2 ml)
  - MM (if possible min. 1 ml) – Breast milk syringe or 15 ml Falcon
  - 1 stool tube (sufficient portion for a total of 3 aliquots to be generated in the laboratory)

Swabs are stored on ice and samples are transferred to the laboratory as quick as possible for processing or biobanking.

- The **documentation of the basic data** is carried out by the study nurse using RedCap on the basis of CRF1 and eCRF1

The auxological measures of the physical examination are documented in the CRF1.

- The **MIAI ID** is continuously generated from the list *1_Proben_pro_Proband_MIAI* (in metadata folder AGV).
- The sample receipt and basic data are then transferred to the *0_Probenliste_METADATEN* and *1_Proben_pro_Proband_MIAI* lists. In this case, each sample receipt of a test subject receives a sampling ID (see SOP Database Management).

**V. Physical examination and sampling d30 and LM6 in study outpatient clinic**

- Telephone contact to make an appointment
- Parents are asked to bring the maternity record, the doctor’s check up-booklet („gelbes Heft“), vaccination certificate and a fresh stool sample to the appointment
- **Physical examination and sampling at d30 or life month (LM) 6** in the study outpatient clinic of the UKW (in case of emergency, home sampling by parents is also possible (see separate SOP))
- **Biosamples on d30 or LM6** (order: swabs, MM, stool): - 2x skin swabs (sternum) – moisten swab for swab A in submitted PBS+BSA, swab sternum and in Eppendorf tube with PBS+BSA; Swab for swab B from moistened skin on sternum and then dry in Eppendorf tube
  - 3x throat swab – Swab A in Eppendorf tube with PBS+BSA presented, swabs B and C in an Eppendorf tube
  - human milk (if possible min. 1 ml) – human milk syringe or 15 ml Falcon tube
  - 1 stool tube (sufficient portion for a total of 3 aliquots to be generated in the laboratory)

Swabs are stored on ice and samples are transferred to the laboratory as quickly as possible for processing or biobanking.

- The **follow-up data** **is documented** by the study nurse using RedCap on the basis of CRF2 or CRF3 and eCRF2 or eCRF3

Physical exam measures are documented in the CRF2 or CRF3.

- Sample receipt and follow-up data are then transferred to the *0_Probenliste_METADATEN* and *1_Proben_pro_Proband_MIAI lists*.

**VI. Physical examination and sampling LM12 in study outpatient clinic**

- Telephone contact to make an appointment
- Parents are asked to bring the check up-booklet, vaccination certificate and a fresh stool sample to the appointment
- **Physical examination and sampling of LM12** in the study UKW outpatient clinic
- **Biosamples on LM12** (order: swabs, nasal brush, BE, MM, stool):

- 2x skin swabs (sternum) – moisten swab for swab A in submitted PBS+BSA, swab sternum and in Eppendorf tube with PBS+BSA; Swab for swab B from moistened skin on sternum and then dry in Eppendorf tube
- 3x throat swab – swab A in Eppendorf tube with PBS+BSA presented, swabs B and C in one Eppendorf tube
- Nasal brush (1 brush per nostril -> put both in the same prepared 5ml EPPENDORF TUBE )- Serum (mind. 0,5ml)
- EDTA-Blood (min. 2 ml)

- human milk (if still breastfeeding min. 1 ml) – human milk syringe or 15 ml Falcon
- 1 stool tube (sufficient portion for a total of 3 aliquots to be generated in the laboratory)

Swabs are stored on ice and samples are transferred to the laboratory as quickly as possible for processing or biobanking.

- The **follow-up data is documented** using CRF4 or eCRF4 by the study nurse using RedCap

The physical measures are documented in the CRF4.

- Sample receipt and follow-up data are then transferred to the *0_Probenliste_METADATEN* and *1_Proben_pro_Proband_MIAI lists*
