# Supplementary material for: A clinical protocol for a German birth cohort study of the Maturation of Immunity Against respiratory viral Infections (MIAI)
Source: Front Immunol. 2024 Sep 17;15:1443665. doi: 10.3389/fimmu.2024.1443665 (PMC11442434; doi:10.3389/fimmu.2024.1443665)
Supplement: Supplementary file 2 [file Table2.docx]

1. **Processing Cord blood**

**DAY 1: Isolation cord blood mononuclear cells (CBMCs) and Monocytes and Cryopreservations**

**Plasma extraction:**

- Centrifuge 100-300 µl of the blood sample at 2000 x g and 4°C for 15 min
- Remove the plasma supernatant and freeze at -80°C
- Biobanking: Enter samples in the SERUM/PLASMA and metadata list (including storage location)!
- Database: Enter the date of sample collection and sample ID in the MIAI list.

**Isolierung of the CBMC´s:**

- Dilute the remaining blood sample immediately 2 (=1/1) with RPMI 1640 (R0883-500ML, Sigma) (without additives) and store at room temperature until processing.
- PBMCs are isolated depending on the blood volume in accordance with the current versions of the following SOPs:
  - **More than 5 ml 🡪 Maxi-Ficoll** 🡪 SOP monocytes_Isolation per Maxi-Ficoll
  - **> 1 ml – 5 ml** **🡪 Midi-Ficoll** 🡪 SOP monocytes_Isolation per Mini o. Midi-Ficoll
  - **<= 1 ml** **🡪 Mini-Ficoll** 🡪 SOP monocytes_Isolation per Mini o. Midi-Ficoll
- Divide the isolated peripheral blood mononuclear cell (PBMC) suspension with the following cell numbers into Falcon tubes:
  a) Monocyte-Isolation 🡪 if possible 3 x 10^7 PBMCs
  b) CBMC-Backup-Aliquot: Cryopreservation of residual CBMCs
- Centrifuge all split PBMC suspensions (500g, 10min room temperature (RT), (ACC9/DEC9), discard supernatants and process cell pellets according to the following SOPs:
  a) SOP Monocytes_Isolation per Maxi/Midi/Mini-Ficoll
  b) SOP_Cryopreservation
- Enter samples in the PBMC preparations list (including storage locations)!
- Enter the date of sample collection and sample ID in the MIAI list.

**Isolation and cultivation of CB monocytes:**

- Depending on the initial blood volume, perform according to SOP Monocyte_Isolation per Maxi/Midi/Mini-Ficoll.
- Set monocyte cell concentration to 1 x 10^6 / ml.
- Seed cells in a96well format (round bottom, suspension)
- Monocyte treatment according to the following work plan:
  Control (Ctrl): 1h PBS_23h Medium
  To do this, 1h after adding PBS, centrifuge the plate (300g, 5 min, RT), washing the cells with warm PBS, centrifuge (300g, 5 min, RT) and carefully resuspend the cells in monocyte medium (same volume as for seeding, no cell counting) and cultivating until the following day.

**Cryopreservation of non-monocytes from the Magnetic Cell Separation (MACS) columns:**

- Rinse column 3 times with 1 ml wash buffer and collect eluate in 15 ml Falcon tubes
- Centrifugation 500g, 10min, RT
- Freeze pellet according to SOP cryopreservation 🡪 Resuspension of 2 x 10^6 MACS cells (=number of PBMCs - number of isolated monos) / ml freezing medium
- Enter cryosamples in the MACS (Biobanking Non-monocytes) column preparations list (including storage locations)!
- Enter the date of sample collection and sample ID in the **MIAI list**.

**DAY 2: Harvesting of monocytes**

- **Harvest from 96well plate for RNA lysates and SNT**:
  - Prepare RA1 lysis buffer (see SOP Preparation of RNA lysates):
   ≥1x10^6 monocytes: 350 µl RA1 + 3,5 µl ßME
   <1x10^6 monocytes: 100 µl RA1 + 2 µl TCEP
  - Joint slab (300g, 5 min, RT)
  - Transfer SNT to labeled Eppendorf tubes -> -80°C
  - If a setting was sown over several wells, the cells are pooled in a 15 ml Falcon. Resuspend cells, harvest and transfer to Falcon. Rinse well with PBS 🡪 also transfer to Falcon. Glaze Falcon (300g, 5 min, RT), discard supernatant.
  - With only a few wells per setting, the cells can be lysed directly in the plate.
  - Lyse the cell pellet in the Falcon or cells in the well with supplemented RA1 buffer well
  - Transfer lysate to labeled Eppendorf tubes -> -80°
- Enter the experiment or generated samples in the list Preparations Monos (tab RNA lysates and SNTs) (including storage location)!
- Enter the date the test was carried out and the test ID in the MIAI list.

1. **processing skin swabs**

- 2 skin swabs (jugulum) HA-A and HA-B sampled and stored on ice until further processing
- Before sampling, 500 µl sterile PBS + 10% BSA were added to the Eppendorf tubes **HA-A**
- Nothing was added to Eppendorf tubes **HA-B**
- Classify Eppendorf tubes s with **HA-A** and **HA-B** in the -80 freezer
- Enter the samples in the **HA smear list** and in the **metadata list** (including storage location)!
- Enter the date of sample collection and sample IDs (e.g. HA-A_MIAI-ID_Lebenstag) in the MIAI list.

1. **processing throat swabs**

- 3 throat swabs **RA-A**, **RA-B** and **RA-C** sampled and stored on ice until further processing
- Before sampling, 250 µl steriles PBS + 10% BSA were placed in the Eppendorf tubes **RA-A**
- Nothing was submitted in the Eppendorf tubes **RA-B +** **RA-C**
- Incubate **RA-A** for 15 min at 4°C on shaker (4°C room). Then store at -80°C together with **RA-B** + **RA-C**.
- Enter the samples in the **RA smear list** and in the **metadata list** (including storage location)!
- Enter the date of sample collection and sample IDs (e.g. RA-A_MIAI-ID_Lebenstag) in the **MIAI list**.

1. **processing nose brushes**

- Two nose brushes are sampled in 5 ml Eppendorf tubes s with **2 ml of isolation medium** (=MEM medium) and stored at RT or in a 37° incubator until further processing
- Nasal brushes are processed as quickly as possible in accordance with the **SOP Airway Epithelial Cell Culture**
- Use directly expanded AECs (d28) for MIAI-ALI test
- Transwell seeding plan for MIAI-ALI trials:
  1. ALI_ZZ (for ZZ and FACS Basic marker panel)
  2. Ctrl: 1h ALI-Medium_**23**h Medium
  3. IAV: 1h IAV 1MOI_**23**h Medium
  4. RSV: 1h RSV 1 MOI_**23**h Medium
  5. RSV: 1h RSV 1 MOI_**47**h Medium
  [1h after virus or HBSS inoculation Wash cells well in 3rd-5th and continue culturing under ALI, see also SOP].
- Enter the samples in the **AEC preparation list (cultivation, Iso-SNT, RNA, SNTs)** and in the **metadata list** (column Obtained biomaterials ->AEC-ID)!
- Enter the date of sample collection and sample IDs in the **MIAI list**.

1. **processing serum sample**

- Centrifuge serum tubes softly at 800 g and RT for 10 min
- Using a 100µl pipette, measure the serum volume (clear, yellowish supernatant), transfer to an Eppendorf tube and freeze at -80°C
- Enter the sample in the **serum list** and in the **metadata list** (including storage location)!
- Enter the date of sample collection and sample ID (Serum_MIAI-ID_Sampling time) in the MIAI list.

1. **processing EDTA blood sample**

**DAY 1: Isolation PBMCs and monocytes, Cryopreservations and SCENITH**

**Plasma extraction:**

Only if no serum sample was obtained from the individual a plasma sample from the EDTA should be collected

- Centrifuge 100-300 µl of the blood sample at 2000 x g and 4°C for 15 min
- Remove the plasma supernatant and freeze at -80°C
- Enter samples in the SERUM/PLASMA and metadata list (including storage location)!
- Enter the date of sample collection and sample ID in the MIAI list.

**Isolation of PBMC´s:**

- Dilute the remaining blood sample immediately 2 (=1/1) with RPMI 1640 (R0883-500ML, Sigma) (without additives) and store at room temperature until processing.
- PBMCs are isolated depending on the blood volume in accordance with the current versions of the following SOPs:
  - **> 1 ml – 5 ml** **🡪 Midi-Ficoll** 🡪 SOP Monocytes_Isolation per Mini o. Midi-Ficoll
  - **<= 1 ml** **🡪 Mini-Ficoll** 🡪 SOP Monocytes_Isolation per Mini o. Midi-Ficoll
- Divide the isolated PBMC suspension into Falcon tubes with the following cell numbers:
  a) Monocytes-Isolation 🡪 if possible 3 x 10^7 PBMCs
  b) SENITH-Analysis 🡪 2 x 10^6 PBMCs
  c) CBMC-Backup-Aliquot: Cryopreservation of residual CBMCs
- Centrifuge all split PBMC suspensions (500g, 10min RT (ACC9/DEC9), discard supernatants and process cell pellets according to the following SOPs:
  a) SOP Monocytes_Isolation per Midi/Mini-Ficoll
  b) SOP_SCENITH_1a_Cukture PBMCs (**CAVE**: Include PBMCs here in RPMI medium w/o Strep)
  c) SOP_Cryopreservation
- Enter the experiment in the **PBMC preparations list**
- Enter the date the test was carried out and the test ID in the **MIAI list**.

**Isolation and cultivation of monocytes:**

- Performance according to SOP Monocyte_Isolation by Mini-Ficoll+MACS
- Set monocyte cell concentration to 1 x 10^6 / ml
- Seed cells for 3 settings (control, Influenza A Virus (IAV) (stock:**3 x 10^7 pfu/ml**), Respiratory-Syncytial Virus (RSV) (stock:**1.2 *10^7pfu/ml**)) in 96well format (round bottom, suspension)
- Monocyte treatment according to the following work plan:
  1) Ctrl: 1h Rest_1h PBS_23h Medium
  2) IAV: 1h Rest_1h IAV 1Multiplicity of Infection (MOI)_23h Medium
  3) RSV: 1h Rest_1h RSV 1 MOI_23h Medium
  For this purpose, 1 h after virus or PBS inoculation, centrifuge the plate (300 g, 5 min, RT), wash the cells with warm (!) PBS, centrifuge (300 g, 5 min, RT) and carefully (!) resuspend the cells in monocyte medium (same volume as for seeding, no cell counting) and continue culturing until the following day.

**Cryopreservation of non-monocytes from the MACS columns:**

- Rinse column 3 times with 1 ml wash buffer and collect eluate in 15 ml Falcon tubes
- Centrifugation 500g, 10min, RT
- Freeze pellet according to SOP cryopreservation 🡪 Resuspension of 2 x 10^6 MACS cells (=number of PBMCs - number of isolated monos) / ml freezing medium
- Enter cryo samples in the **MACS column preparation list** (Biobanking Non-monocytes) (including storage locations)!
- Enter the date of sample collection and sample ID in the **MIAI list**.

**DAY 2: Infection and harvesting of monocytes**

- **Harvest from 96well plate for RNA lysates and SNT**:

- produce RA1-Lysepuffer (see SOP Manufacture RNA-Lysate):
 ≥1x10^6 monocytes: 350 µl RA1 + 3,5 µl ßME
 <1x10^6 monocytes: 100 µl RA1 + 2 µl TCEP

- Centrifuge plate (300g, 5 min, RT)
- Transfer SNT to labeled Eppendorf tubes -> -80°C
- If a setting was seeded over several wells, the cells are pooled in a 15 ml Falcon. Resuspend cells, harvest and transfer to Falcon. Rinse well with PBS -> also transfer to Falcon. Glaze Falcon (300g, 5 min, RT), discard supernatant.
- With only a few wells per setting, the cells can be lysed directly in the plate.
- Lyse the cell pellet in the Falcon tube or cells in the well with supplemented RA1 buffer well
- Transfer lysate to labeled Eppendorf tubes -> -80°

- Enter the experiment or generated samples in the Preparations Monos list (RNA lysates and SNTs tab) (including storage location)!
- Enter the date the test was carried out and the test ID in the **MIAI list**.

1. **processing of expressed human milk samples**

- Centrifuge humanmilk for 10 min at 350 x g at RT
- Remove the aqueous phase (under the fat layer) and transfer to three prepared 2 ml Eppendorf tubes s **MM-A**, **MM-B** and **MM-C** and freeze at -80°C
- Enter the sample in the **human milk list** and in the **metadata list** (including storage location)!
- Enter the date of sample collection and sample IDs (e.g. MM-A_MIAI-ID_Sampling time) in the **MIAI list**.
- Attention for humanmilk from multiples: create a separate line with a separate sampling ID for each child in the metadata list and assign species-specific MIAI IDs for the breast milk -> MM IDs receive the suffix “-M” for “multiples” (-> MIAI-ID_Sampling- Time-M)

1. **processing expressed human milk with BMC isolation**

- Centrifuge breast milk for 20 min at 800 x g at RT
- Remove fat layer and supernatant, transfer and centrifuge suspension again (10 min and 350 x g at RT) -> remove supernatant under fat layer, transfer to three prepared 2 ml Eppendorf tubes s **MM-A**, **MM-B** and **MM-C** and freeze at -80°C
- Resuspend cell pellet in 1 ml cold PBS, transfer and add another 4 ml PBS -> centrifuge for 10 min at 500 x g at 4°C
- Discard supernatant and resuspend cell pellet in 1-3 ml cold PBS and transfer as fat-free as possible into a new 15 ml Falcon. If the cell pellet is large, top up with 1-3 ml cold PBS.
- Counting cells using a Neubauer chamber

for < 2x10^6^ cells -> 1 Aliquot

for 2-4 x 10^6^ cells -> 1 Aliquot à 2 x 10^6^ cells for Sequencing + 1 Aliquot with remaining

cells für FACS

for > 4 x 10^6^ cells -> min. 2 Aliquots à 2 x 10^6^ cells

- Centrifuge cells: 500 x g, 10 min, 4°C -> discard supernatant
- Resuspend cells in ice-cold freezing solution (FCS + 10% DMSO), transfer immediately to cryotubes (on ice) and freeze at -80°C using a freezing carousel
- After approx. 24 hours, transfer the cryotubes from the freezing carousel to the appropriate storage box (on dry ice) and store at -152°C.
- Enter data (ID, sample volume, cell count and storage details etc.) in the **sample list_humanmilk** and in the **metadata list**!
- Transfer date of sampling and sample ID to **1_Samples per subject_MIAI list**

1. **processing of stool sample**

- Sample from stool tubes is divided into three Eppendorf tubes s **FC-A**, **FC-B** and **FC-C**
- 600 µL DNA/RNA Shield is added to the FC-B sample
- Freeze samples at -80°C
- Enter the samples in the **stool** and **metadata list** (including storage location)!
- Enter the date of sample collection and sample IDs (e.g. FC-A_MIAI-ID_Sampling time) in the **MIAI list**.

**Attention!** 600 µL of DNA/RNA Shield has already been added to tubes for home sampling. Vortex the tube with inhibitor for the B sample vigorously before transferring the sample to Eppendorf tubes to mix the stool and inhibitor!
